# Passive limitation of surface contamination by perFluoroDecylTrichloroSilane coatings in the ISS during the MATISS experiments

**DOI:** 10.1038/s41526-022-00218-3

**Published:** 2022-08-04

**Authors:** Laurence Lemelle, Sébastien Rouquette, Eléonore Mottin, Denis Le Tourneau, Pierre R. Marcoux, Cécile Thévenot, Alain Maillet, Guillaume Nonglaton, Christophe Place

**Affiliations:** 1 ENS de Lyon, CNRS, Laboratoire de Géologie de Lyon-Terre Planètes et Environnement, 46 allée d’Italie, F-69342 Lyon, France; 2 CNES, 18 Avenue Edouard Belin, 31401 Toulouse, Cedex 9 France; 3 ENS de Lyon, CNRS, Laboratoire de physique, 46 allée d’Italie, F-69342 Lyon, France; 4 Université Grenoble Alpes, CEA, LETI, DTBS, 17 rue des Martyrs, 38000 Grenoble, France; 5 MEDES-IMPS for CADMOS, Waypost-4e étage, 2 Av. de l’Aérodrome de Montaudran, CS77720, BP 74404, F-31405 Toulouse, Cedex 4 France

**Keywords:** Aerospace engineering, Biophysics

## Abstract

Future long-duration human spaceflight will require developments to limit biocontamination of surface habitats. The MATISS (Microbial Aerosol Tethering on Innovative Surfaces in the international Space Station) experiments allowed for exposing surface treatments in the ISS (International Space Station) using a sample-holder developed to this end. Three campaigns of FDTS (perFluoroDecylTrichloroSilane) surface exposures were performed over monthly durations during distinct periods. Tile scanning optical microscopy (×3 and ×30 magnifications) showed a relatively clean environment with a few particles on the surface (0.8 to 7 particles per mm^2^). The varied densities and shapes in the coarse area fraction (50–1500 µm^2^) indicated different sources of contamination in the long term, while the bacteriomorph shapes of the fine area fraction (0.5–15 µm^2^) were consistent with microbial contamination. The surface contamination rates correlate to astronauts’ occupancy rates on board. Asymmetric particles density profiles formed throughout time along the air-flow. The higher density values were located near the flow entry for the coarse particles, while the opposite was the case for the fine particles, probably indicating the hydrophobic interaction of particles with the FDTS surface.

## Introduction

Long-duration human spaceflights, like missions to Mars^1,2^, and those involving a continuous presence in low Earth orbit (LEO), require reconsideration of risk management, in particular of biohazardous risks to astronauts and equipment integrity^2–9^. Over long periods, unknown mutations, resistance, and virulence can develop and be favored in response to recurrent disinfectant application and adaptation to microgravity^10–21^. Besides, biocontamination of spacecraft and in particular their surfaces^22–25^ by crew flora^3,26,27^ is inevitable^5,28,29^. As sources of holders, the surfaces in the ISS may retain and favor microbial biofilms in which microorganisms are protected from inhospitable environmental variations and from killing by antibiotics and disinfectants^5,30–33^. In this respect, surfaces then become infection *foci* and transmission routes of pathogens by contact^34–40^. Equipment degradation due to surface corrosion related to microbial metabolic activities observed on MIR and in the early days of the ISS (International Space Station) are also sources of concern^34,36^.

The development of smart surface designs with optimized performances in microgravity is part of a strategic upstream step for constructing novel spacecrafts for long-term exploration^41^. The selection of advanced surfaces that do not allow microbes to stick and grow over large areas, effectively making them easier to clean and more hygienic, is a prerequisite. Among this set of surfaces, evaluating bacterio-repellent or bacteriostatic surfaces already implemented in numerous industrial fields that do not require demonstrating innocuity to astronaut health, appears to be a pragmatic approach. Under microgravity, infectious agents are conveyed by aerosols, as on Earth, but also by much larger water droplets and floating condensates^24,42^. Evaluating hydrophobic coatings that limit the surface contamination by repulsing water droplets and floating condensates, prior to any microbial species-dependent interaction, appears to be a promising approach^43^. Simulations under microgravity, and in the specific environment of ISS modules, have not yet been validated experimentally, although fluid dynamics computations are efficient in deciphering parameters and trends of bioaerosol contamination in an enclosed space^44^. Testing the ISS’s true bioaerosol contamination on surfaces still requires exposing them in situ in the ISS.

The experiment MATISS (Microbial Aerosol Tethering on Innovative Surfaces in the International Space Station) was conceived to this end. It was based on the use of a sample holder developed to expose, over the long term in the ISS, a glass lamella surface, without risks and with simple handling for the astronauts (Supplementary Fig. 1), and aimed to investigate the surface contamination, once returned to the laboratory, across the sealed holder. Such designed non-invasive and non-destructive approach preserves the possibility of extending this particulate characterization in the future by other approaches informative of the chemical and microbiological compositions of the contamination. During the MATISS-1 campaign, a proof of concept demonstrated its usefulness for investigating the efficiency of hydrophobic coatings^45^. Hydrophobic surface coatings and an untreated glass surface were exposed for 6 months. In the ISS, hydrophobic coatings can be mainly covered by lipids and debris. The absence of iridescent organic patches and the low value of few particles per mm^2^ detected on the hydrophobic coatings after six months of exposure sets the potential efficiency of the hydrophobic coatings over multi-years lifetimes of spacecraft’s cabins, in particular in low touch area. Besides, twice as many large particles accumulated on the organofluorosilane coating (FDTS) than on the others. However, the opposite trend was observed for fine particles, suggesting the FDTS coating as our most efficient hydrophobic coating to prevent surface contamination by microorganisms or small colonies carried by hydrophilic droplets.

The usefulness of fluoroalkyl silanes, and more particularly of FDTS, to reduce the surface energy of a material and thus reduce the adsorption of proteins or bacteria is well documented^46–52^. The FDTS coatings are well-known and relatively cheap organofluorosilane commonly used, alone or in combination with topography, for their intrinsic hydrophobic properties, but also anti-stiction, and passivation properties for a large number of potential applications such as aeronautics, sensors, biosensors^53,54^, microelectronics, microfluidics^55^ and textiles^56^. In addition, commercialized industrial equipment can process FDTS on large surfaces and achieve high-volume production with good reproducibility. It is noteworthy that previous cytotoxicity studies demonstrated the nontoxicity^57^ of fluorosilane-based coatings. Furthermore, an FDTS coating has already been used to improve blood biocompatibility^58^ and for the fabrication of wearable and implantable medical devices. The existing industrial processes of FDTS deposition summarized above support the choice in this study to work with FDTS to establish if and how this coating limits biocontamination in the international space station. It has the advantage of ensuring upstream the possibility of developing processes to produce durable hydrophobic coatings on space habitat construction materials to limit the biocontamination of surface habitats. Such proof of concept would afterward support the study of a larger panel of hydrophobic coatings in the perspective of industrialization.

Therefore, the MATISS campaign has been triplicated to expose FDTS coating’s over several months in the ISS: from November 2016, for ∼6 months for the MATISS-1 campaign; from August 2018 for ~1, ~3, and ~12 months for the MATISS-2 campaign and from September 2019 for ∼12 months for the MATISS-2.5 campaign (Material and Methods). In this study, an FDTS coating’s impact on the surface biocontamination was established following the spatial spreads of the bioaerosols and their progress versus time on a FDTS-coated glass lamella and control surfaces. The sources and routes of the surface biocontamination in the ISS and the role of hydrophobicity on such biocontamination are discussed.

## Results

### Exposure of FDTS surfaces to the Columbus atmosphere

FDTS coating were prepared with an aliphatic silane that was applied by molecular vapor deposition on silica glass lamella (Material and Method). These molecules form self-assembled monolayers covalently bound to the hydroxyl groups of the silica glass surfaces, obtained by a silanization method^59^. A new batch was prepared for each campaign, and its properties checked in order to guaranteeing that coatings themselves are not a parameter of the FDTS contamination in the three campaigns. The value of the optical thickness of the coatings (Material and Method) was 1.6 ± 0.2 nm (Average ± precision, for 3 substrates) and is reproducible from batch to batch (SD = 0.2 nm for 3 batches). It is consistent with other values reported in the literature^53^ and with the theoretical length value of approximately 1.4 nm of an FDTS molecule^60^. The fluorinated molecules have a low surface energy and adopt a preferential orientation with the fluorinated tails towards the coating-air interface^56,61^. The roughness of FDTS coatings is negligible and was 0.32 ± 0.07 nm (Average ± precision, for 6 measurement series). The resulting high water contact angles values (Material and Method) was equal to 110 ± 2° (Average ± precision) with a variability from one batch to another lower than 2° (SD).

The FDTS coatings were exposed in the Columbus module, using a previously developed sample holder (Fig. 1 in Lemelle et al., 2020^45^). A lateral 2-mm–thick slit in the holder allows the circulation on the coatings of a laminar airflow of velocity value in the range of 10–40 ft/min, as evaluated considering an air exchange of the atmosphere ensured by a flow rate value of ca. 400 m^3^/h.

**Fig. 1:**
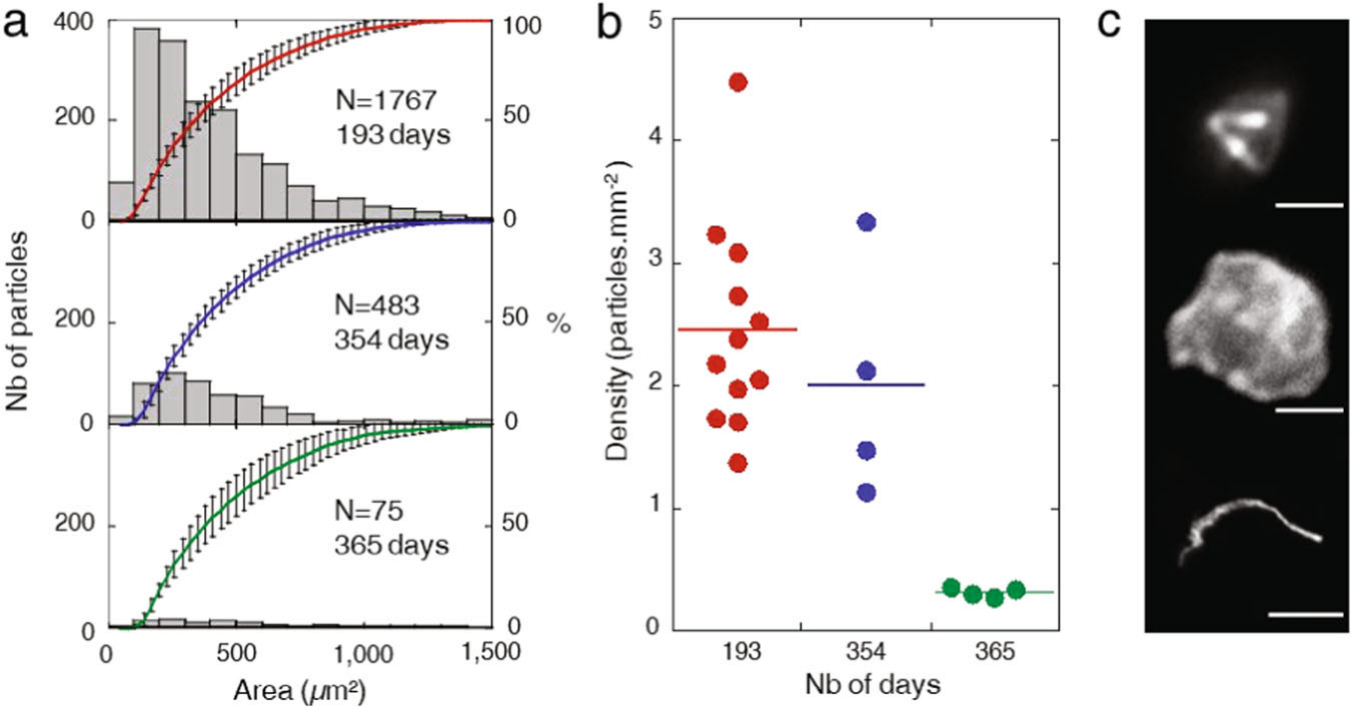
Surface contamination kinetics by coarse particles (50 < Area < 1500µm^2^) on FDTS coating. **a** Particle size distribution histogram (grey bars) versus the area in μm² and cumulative particle size curves after MATISS-1 (193 days of exposure (red)), MATISS-2 (354 days (blue)) and MATISS-2.5 (365 days (green)) campaigns. Each holder has its symbol. Error bars represent the standard deviation of the data. **b** Dot-plots showing the density values of the coarse particles measured on surfaces of 8 x 8 mm. Centerlines show mean values: 2.45 ± 0.81 for MATISS-1 (193 days), 2.01 ± 0.84 for MATISS-2 (354 days) and 0.36 ± 0.12 for MATISS-2.5 (365 days). **c** Mosaic of optical images recorded at low magnification displaying typical shapes of coarse particles (top and middle, scale bar is 10 μm) and macroscopic particles (area > 1500 μm², bottom, scale bar is 100 μm).

The biocontamination in the ISS of the external surfaces of the holders containing the FDTS coated lamellae were checked upon the reception day in the laboratory. Numerous clones were cultivated from all the microbial swabbings of the probed holders (Material and Methods). The long lag times before starting exponential growth of these microbial cultures (up to 5 days) revealed stressed states anticipated for microorganisms likely deposited few months ago in the ISS. The taxonomy of most of the identified isolates showed mostly gram-positives and also provided not unexpected insight into their microbial ecology (Materials and Methods and Supplementary Table 1). They belong to three phyla: *Firmicutes* (gram-positive), *Actinobacteria* (gram-positive), and *Proteobacteria* (gram-negative). Isolated genera can be characteristic of the normal microbiota of skin and mucous membranes (*Staphylococcus*, *Micrococcus*, *Bacillus*), of the outer ear and the intestine (*Pseudomonas*)^62,63^. Alternatively, they are rather characteristic to soil and/or water habitats: *Pseudarthrobacter* and *Arthrobacter, Bacillus* and *Paenibacillus*^64,65^. Besides, some of the isolated species had not been signaled yet, such as *Pseudomonas fulva*, *Paenibacillus sp*. or *Pseudarthrobacter sp*. most are reported in the survey of the environmental biocontamination of the ISS^22^, such as *Staphylococcus hominis* or *Micrococcus luteus*.

Then the position, area, shape, and intensity of all the coarse and fine particles (area value >50 µm^2^ and <50 µm^2^ respectively) on the FDTS coated lamellae and the controls were measured by tile scanning optical microscopy applied at two different magnifications, ×3 and ×30 (Material and Method).

### Long term particle contaminations of the FDTS coating

The size distributions and the corresponding cumulative curves of the coarse particles on the FDTS coated lamellae exposed during the three MATISS campaigns are presented in Fig. 1a. They display quite comparable monomodal distributions of particles with area values in the range of 50 µm^2^ to 1500 µm^2^, and with the most probable size equal to approximately 155 µm^2^. However, the particle density values observed for the longest exposure times from one campaign to another are different (Fig. 1b). The mean average density value is centered around fewer than 2 particles per square millimeter after 193 days for the MATISS-1 campaign and after 354 days for the MATISS-2 campaign, but less than 1 particle per square millimeter after 365 days for the MATISS-2.5 campaign. These differences, not proportional to the exposure time gaps, displays a large variability of the particle loads of the air in the ISS during the three campaigns. The similarity of the size distributions (Fig. 1a and Supplementary Fig. 2a) may suggest an invariant coarse particle load over time. This is refuted by a systematic scan of the shapes of the particles collected during each campaign. Flat hexagonal particles consistent with scale discs (tissue or skin) and fibrillar particles (Fig. 1c bottom, and Supplementary Fig. 2 in Lemelle et al. 2020^45^) in the MATISS-1 campaign were overrepresented compared with the round or angular particles in the MATISS-2 and MATISS-2.5 campaigns (Supplementary Fig. 3). In comparison, very few particles larger than 1500 µm were observed. The shapes of the MATISS-2.5 particles are similar to those of MATISS-2 and they have clearly visible edges. Whatever the elongation of the particles, two main types of particles were observed. The first type presents shapes with sharp points, and large surfaces on which the edges are parallel, which suggests crystallographic cleavage planes. A second type presents more parallelepipedic forms, with perpendicular edges, which delimit in hollow and in relief many small cubic volumes, which suggests an aggregate of small crystals or a crystal whose growth is imperfect. Shape diversity indicates a temporal change of the sources of the coarse particle loads in the air in the ISS that are most probably related to the astronauts’ activities.

**Fig. 2:**
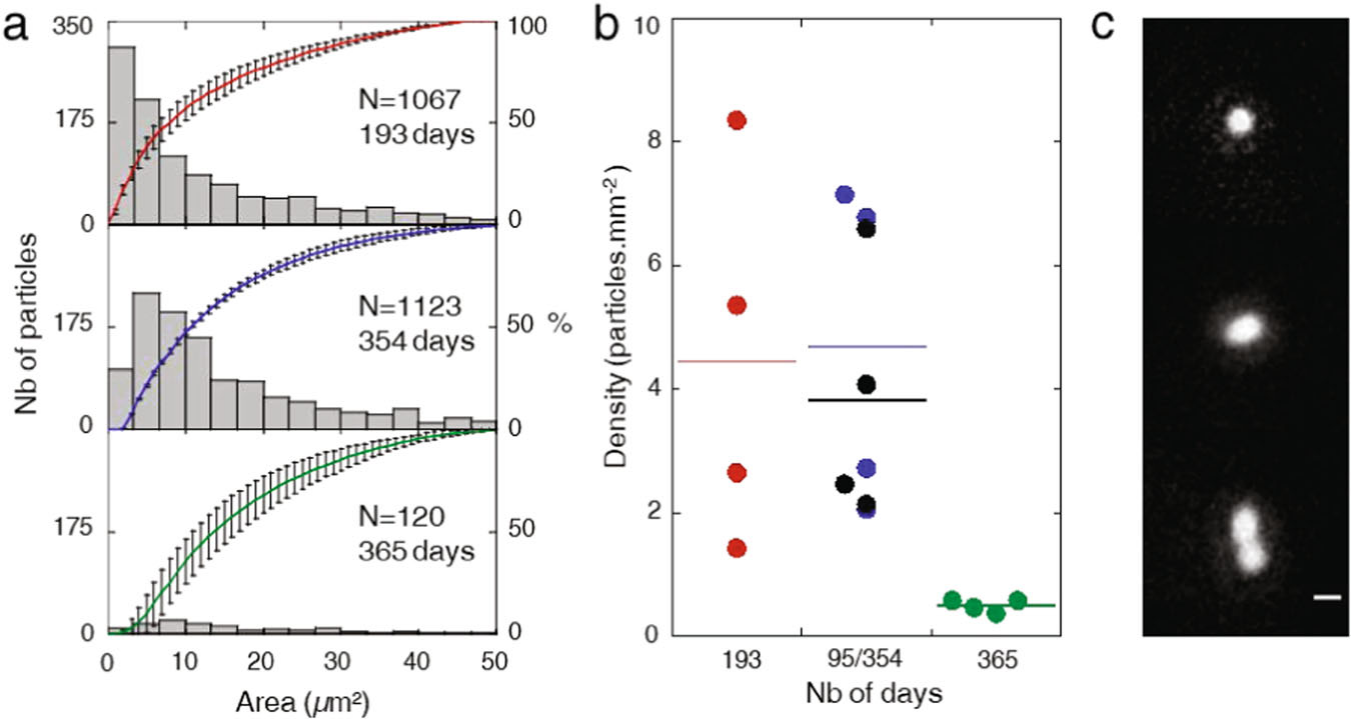
Surface contamination kinetics by fine particles (0.50 < Area < 50 µm^2^) on FDTS coating. **a** Particle size distribution histogram (grey bars) versus the area in μm² and cumulative particle size curves for MATISS-1 (193 days of exposure (red)), MATISS-2 (354 days (blue)) and MATISS-2.5 (365 days (green)) campaigns. Each holder has its symbol. Error bars represent the standard deviation of the data. **b** Dot-plots showing the density values of the fine particles measured on surfaces of 8 x 8 mm. Centerlines show mean values: 4.45 ± 2.66 for MATISS-1 (193 days), 4.68 ± 2.30 for MATISS-2 (354 days) and 1.02 ± 0.58 for MATISS-2.5 (365 days). For MATISS-2, the density values (black) measured on surfaces exposed for 95 days with the same experiment for 354 days are also reported. The black centerline shows a mean value of 3.82 ± 1.77 after 95 days. **c** Mosaic of optical images recorded at high magnification displaying typical shapes of fine particles (scale bar is 2 μm).

The size distributions and the corresponding cumulative curves of the fine particles on the FDTS coated lamellae exposed during the different MATISS campaigns are presented in Fig. 2a. As observed for the coarse particles, the mean values of the particle densities are centered around a similar average density value of fewer than 4 particles per square millimeter, after 193 days for the MATISS-1 campaign and after 354 days for the MATISS-2 campaign. This value is much higher than the 1 particle per square millimeter measured after 365 days for the MATISS-2.5 campaign (Fig. 2b). This density gap confirms the variability of the loads of the air in the ISS over the long term for the fine particles, mainly due to dissimilar fractions of particles in the range of 0 to 20 µm^2^ (Supplementary Fig. 2b). Indeed, the particles of average area consistent with microbial cells (area < 20µm^2^) observed in the three campaigns show either an isolated round shape consistent with those of a single *coccus*, or a round or elongated shape with constriction figures, consistent with division features of filamentous microbial cells or *cocci* (Fig. 2c, Supplementary Fig. 4). Pictures of the fine particles may even display halos that are probably a trace of the evaporation of the water droplets that carried the fine particles in the ISS atmosphere. So, while the intensity of the fine particle contamination varies over the long term, their shape and nature, probably of microbial origin, is persistent over the long term.

### Kinetics of the FDTS surface particle contaminations over the long term

**Fig. 3:**
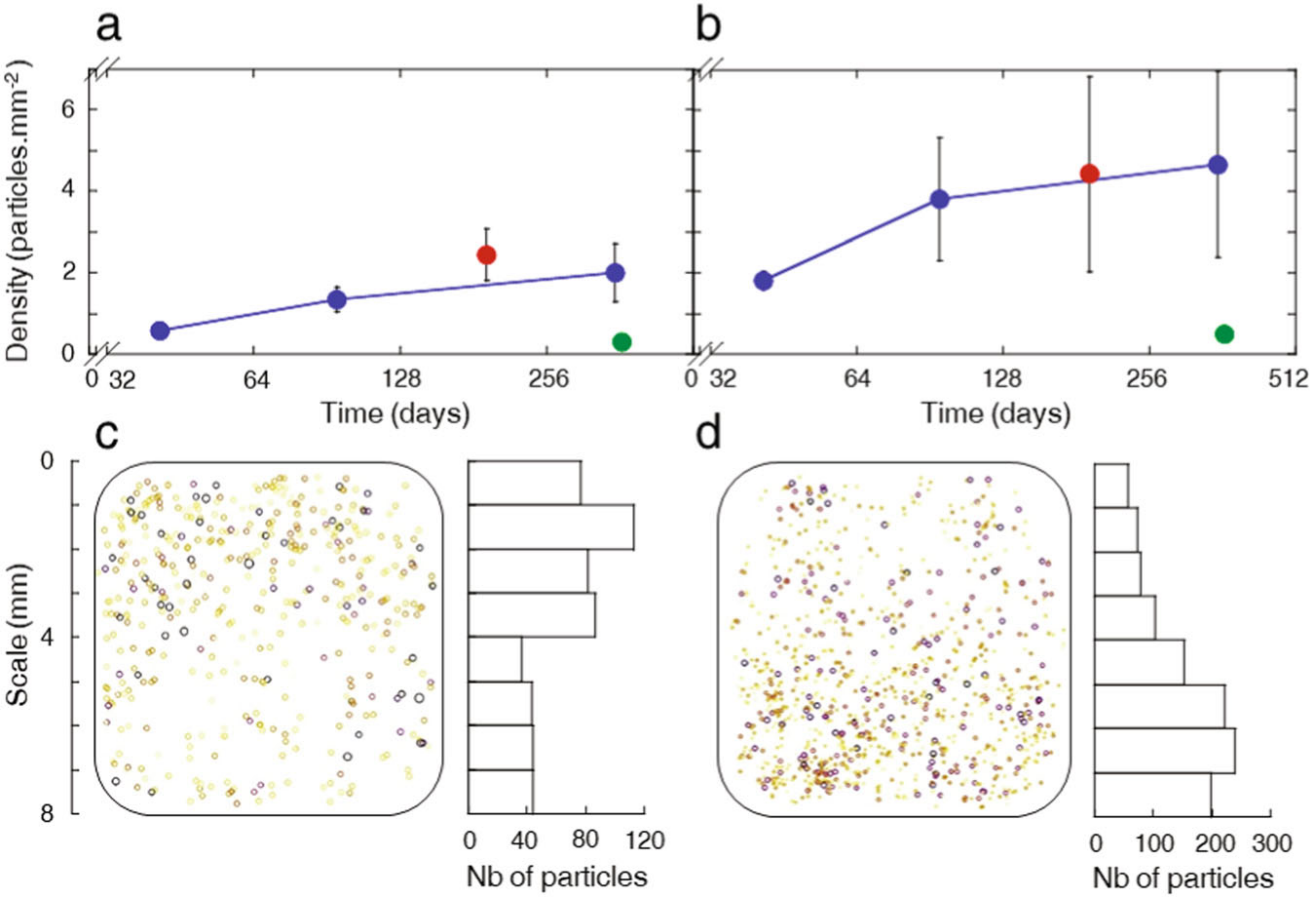
Surface contamination kinetics on FDTS coating in MATISS-2 campaign. **a** Density values of the coarse particles (50 μm^2^ < Area < 1500 μm^2^) measured on surfaces of 8 x 8 mm. **b** Density values of the fine particles (0.50 μm^2^ < Area < 50 μm^2^). The blue curves show the kinetics of the surface contamination increase. Red and green symbols show the FDTS-surfaces exposed for 193 days in MATISS-1 campaign and 365 days in MATISS-2.5 campaign, respectively, in different experimental periods than that of the blue dots. The FDTS-surfaces exposed Matiss-2.5 campaign was deposited on a borosilicate glass lamella (green dot) and suprasil lamella (green triangle). Error bars represent the standard deviation of the data. **c** Schematic representation of the location of the coarse particles on FDTS surfaces within a window exposed for 354 days (left). The side near the closest aperture is in y position =0. Each particle is represented by a circle whose color (yellow/orange/violet scale from 50 to 500 µm²) and sizes (ratio 1 to 1.5 from 50 to 1500 µm²) are proportional to the particle surface. The particles number histogram versus the y position (vertical axis) of particles on surfaces (right). **d** Same representation for fine particles with a yellow/orange/violet scale from 0 to 50 µm² and size ratio of 1 to 1.5 for 0 to 50 µm² particle surfaces.

For the MATISS-2 campaign, average density values are reported versus exposure time (Fig. 3a and b blue) to determine the rate law of the FDTS contamination by the coarse particles (Fig. 3a) and fine particles (Fig. 3b). Sample holders were initially all installed simultaneously, in the same location, and were removed, sealed, and stored periodically, at approximately doubled exposure times. The deposition kinetics of the particles varied with time. Kinetics is not discussed in terms of mechanism since it was shown above to vary over monthly periods mainly due to biocontamination source variation. An almost linear accumulation of coarse particles was then evaluated (Fig. 3a) with a coverage rate value of ∼0.5% per year, consistent with the value previously evaluated^45^. The accumulation rates of fine particles are about 10 times smaller (Fig. 3b). The long-term particle accumulation rates observed in MATISS-2 are consistent with the ones determined for MATISS-1 (Fig. 3a and b, blue and red) while the accumulation rates during MATISS-2.5 being much lower (Fig. 3a and b, blue and green).

A closer inspection was made of the particle distributions formed in the set of the FDTS coated lamellae exposed in the holders to about one year of laminar flows in the MATISS-2 campaign. It is to be noted that these FDTS coated lamellae were always mounted in a position such that the main air-flow enter from one side of the lamella (Supplementary Fig. 1). Particle positions display non-homogeneous particle densities, with asymmetric distributions formed along the air-flow direction, summarized by the histograms. Coarse particles are accumulated close to the aperture (positioned on x = 0) (Fig. 3c), whereas fine particles are far from the holder aperture (Fig. 3d). These asymmetric distributions are progressively formed along the exposure (Supplementary Fig. 5b). They are formed on the FDTS coatings, and not observed on the glass control (Supplementary Fig. 5a). These results display specific particle/hydrophobic surface interactions for the coarse and fine particles that are put into play when the air flows on the lamellae exposed in the MATISS holder (Discussion).

## Discussion

**Fig. 4:**
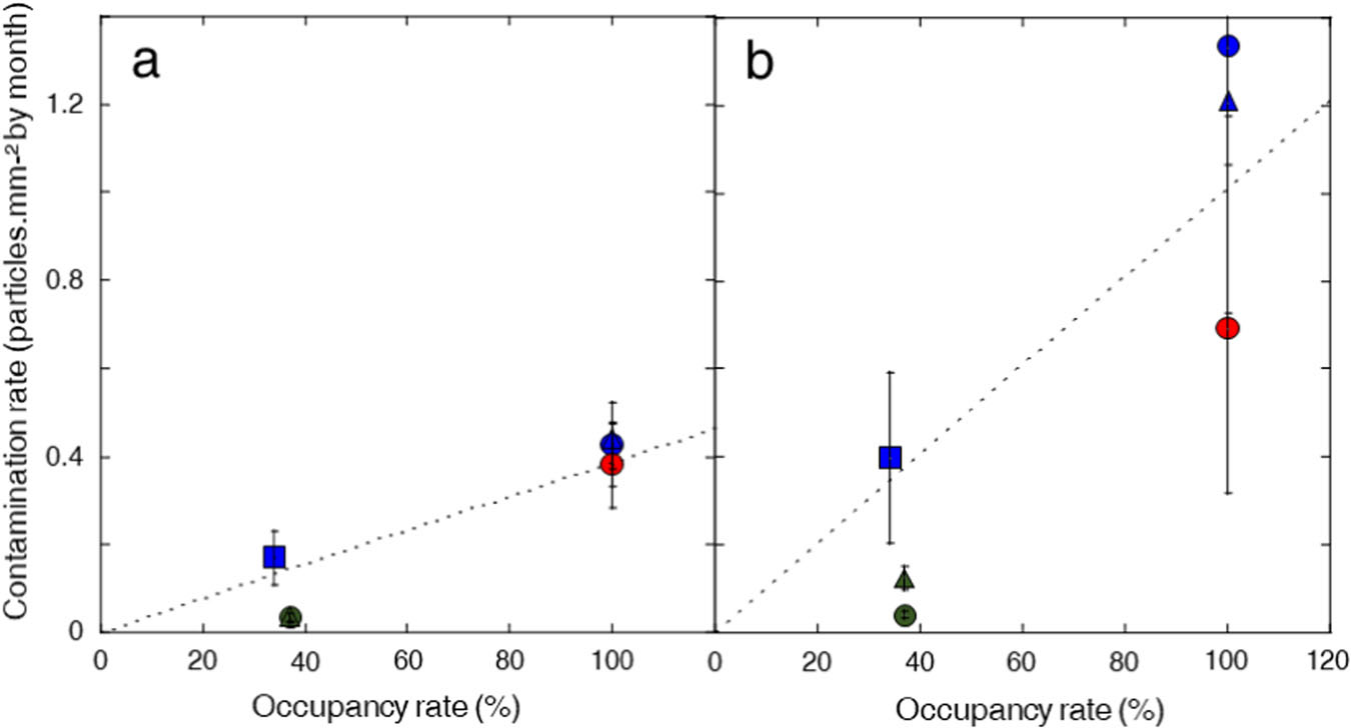
Surface contamination rate (θ) versus Occupancy rate (η). **a** Surface contamination rate values of the coarse particles (50 μm^2^ < Area < 1500 μm^2^). **b** Surface contamination rate values of the fine particles (0.50 μm^2^ < Area < 50 μm^2^). Red and green dots show the FDTS-surfaces exposed for 193 days in MATISS-1 and 365 days in MATISS-2.5, respectively, blue dots for the different experimental periods of MATISS-2 (41 days, 95 days and 354 days). Each holder has its symbol. Error bars represent the standard deviation of the data. The dashed curves are guide-eyes of an increase of the surface contamination rate with the occupancy rate.

In these campaigns that spanned from 2016 to 2020, density values of only a few particles per mm^2^ deposited over a few months were measured initially during MATISS-1. These low values confirm that the surfaces are kept relatively clean in the Columbus module. However, the amount of contamination was shown to vary considerably depending on the campaign. Contamination rate values were similar in the MATISS-1 and MATISS-2 campaigns but lower for fine and coarse particles in the MATISS-2.5 campaign. The thickness, surface roughness, and hydrophobicity of the coatings prepared for the three campaigns being indistinguishable, this difference could be assigned to the activity in the ISS, and most likely related to both the astronauts’ presence and their activities. As a first approximation, it can be hypothesized that the surface contamination depends on the number of astronauts working in the Columbus module. Occupancy rates of the Columbus Module for each campaign, η (%), were evaluated considering only the occupancy periods of the European astronauts (Supplementary Table 2). Their values (η ≈ 35% or 100%) confirm the possibility of contrasted regimes of surface contamination in the different MATISS campaigns. A high regime of surface contamination is indeed observed during the high occupancy rate exposure of MATISS-1 and MATISS-2, and a low regime (η ≈ 35%) over the entire low occupancy rate periods of exposure of MATISS-2 and MATISS-2.5 (Fig. 4). In particular, the lowest MATISS-2.5 contamination rate may be related to the fact that this campaign was conducted during the Covid-19 pandemic, resulting in fewer global astronauts present on board.

Besides this important variation of the number of particles released in the binnacle, the nature of the contaminants was also observed to vary for the coarse particles (more fibers for MATISS-1 and more minerals for MATISS-2 and 2.5). Production of coarse particles can therefore likely be linked to the monthly and specific shuttle maintenance activities of the astronauts on the different building materials present in the spacecraft, rather than on their daily activities. This underscores the need for a highly efficient air filtration in particular during these activities to ensure the removal of large particles that astronauts might otherwise breathe in afterwards. In contrast, the fine particles show some invariance in shape, with numerous bacteriomorphs, as particles divided into sub-particles of similar µm-size, as well as µm-particles with a halo evoking the evaporation of a droplet of water. Measuring surface contamination rates associated with the physical care of the astronauts or with their activity of maintenance of their environment appears to be a promising approach to evaluate whether a low number of very active astronauts or the opposite is the least contaminant approach on long-duration human spaceflights.

In this study, opposite stratifications of coarse particles and fine particles were observed on the FDTS surfaces, with coarse particles deposited near the opening of the holder, and fine particles far from the opening. As this phenomenon was not observed on the control surfaces, it was related to the specific coarse and fine particle/hydrophobic surface interactions (see Results, section 3). The particles are carried along a forced path in the sample holder, by the laminar air-flows that are aspirated in the nearby return grid. The load of particles of the air-flows enters the sample holder only through the lateral sides. The stronger the particle’s affinity with the surface, the closer to the air inlet it will settle, contributing to segregating spatially the particles according to the strength of the interactions with the hydrophobic coating. In this respect, the coarse hydrophobic particles should behave differently to the fine hydrophilic particles. The opposing stratifications of the coarse and fine particles on the FDTS surface are related to their different nature, not to their size. The hydrophobic character of the FDTS surface should capture coarse hydrophobic particles (skin, synthetic fabric,…) near the air inlet, while the fine hydrophilic particles should be repulsed. However, their accumulation at a given distance away from the inlet was unexpected, suggesting a more complex interaction, possibly involving sliding or jumping of water droplets. Such behaviors of sliding or jumping water droplets has already been observed but on superhydrophobic surfaces satisfying the Cassie-Baxter equation^66^. Under microgravity, the multiple contacts are not related to the weight of the droplets and are facilitated by the small contact surface between the droplet and the hydrophobic surface^67^. Multiple interactions between the surface and the droplets could account for the final deposition of the particles^68,69^. Repeated impacts under air-flow lead to droplet modifications, which may modify in return the surface/droplet interaction itself and increase the probability of particle deposition. The farthest distances from the inlet will be the most populated with particles. This property of a hydrophobic surface in microgravity revealed here could be exploited to improve the cleaning of the surfaces in the station^70^.

In conclusion, these observations lay the foundation for developing some rationale for integrating hydrophobic coatings, like FDTS, as a promising approach to passive contamination control into a more comprehensive strategy for cleaning spacecraft. The interest in using hydrophobic surfaces would be, as presupposed, the weaker attachment of the droplets of water, and more precisely the possibility that the droplets jump or slide along the surfaces. The lifetime of a droplet appears here to be a critical parameter because microorganisms from an evaporated droplet should land on the hydrophobic surface, attach to it and eventually contaminate the surface. Such a perspective points to the need to develop new surfaces that not only reduce the surface/droplet interaction, but also reduce the possibility of attachment of microorganisms.

## Materials and methods

### FDTS coatings

FDTS coatings are based on 1H,1H,2H,2H-perfluorodecyltrichlorosilane FDTS (ABCR, 97%). The coating application was performed using commercially available molecular vapor deposition equipment (MVD100 from Applied MST, San José, US). The deposition conditions for FDTS were as follows. In a first step, the surface was cleaned using remote RF oxygen plasma (450sccm O_2_ flow, 250 W, 300 s). In a second step, one cycle of tetrachlorosilane SiCl_4_ (Sigma Aldrich, 99,998% Semiconductor grade) at 18 Torr was injected, followed by four cycles of water at 18 Torr. This step took place for a duration of 600 s at 35 ^◦^C. In a third step, two cycles of FDTS at 0.5 Torr were injected, followed by one cycle of water at 18 Torr. This step took place for a duration of 900 s at 35 ^◦^C and aimed at grafting FDTS to the surface by a salinization reaction. The optical thickness of each FDTS layer was extrapolated from measurement obtained by the Surface Enhanced Ellipsometric Contrast technique on thermally oxidized silicon substrates. The *Rq* roughness measurement was determined on FDTS functionalized oxidized silicon substrates by Dimension Icon atomic force microscope from Bruker. The water contact angle was measured by goniometer equipment from GBX instruments, on each FDTS functionalized glass borosilicate and suprasil slides.

### Sampling with the MATISS sample holder

MATISS sample holders were sterilized with isopropanol and mounted with glass lamellae. They were sealed with Kapton tape, and placed into two Ziploc bags using gloves when not exposed to air. The three sequential MATISS campaigns (MATISS-1, MATISS-2, and MATISS-2.5) brought 7 sample holders into the Columbus module by Cygnus CRS OA-5 on the 17th October 2016, by CRS OA-9 on the 20th May 2018 and by SpX-18 on the 25th July 2019. For the MATISS-1 campaign, two holders were mounted in the direct vicinity of the Return Grid Sensor Housing (Supplementary Fig. 1). One holder was mounted on the surface of the EPM Rack (European Physiology Modules) front panel in the middle section of the Columbus cabin (see Materials and Method of Lemelle et al. 2020^45^). Sample holders were exposed to air on the 21st November 2016 for MATISS-1 and sealed with Kapton tape using gloves, placed into two Ziploc bags, and stored at room temperature the day before the return with the Soyuz 49 S on the 2nd June 2017 after a 193 days exposure. The sample holders of all the other campaigns were installed near the Return Grid Sensor Housing. They were exposed to air on the 23rd August 2018 for MATISS-2 and the 25th September 2019 for MATISS-2.5. For the MATISS-2 campaign, samples were sealed on the 3rd October 2018 and returned with the Soyuz 54 S on the 4th October 2018 after a 41 days exposure, sealed on the 26th November 2018 and returned with the Soyuz 55 S on the 20th December 2018 after a 91 days exposure and sealed on the 12th August 2019 and returned with the SpX-18 on the 31th August 2019 after a 354 days exposure. For the MATISS-2.5 campaign, samples were sealed on the 24th September 2020 and returned with the Soyuz 62 S on the 21th October 2020 after a 365 days exposure. Sample holders transfers to our laboratory were achieved in less than a week, avoiding X-ray scans and using temperature monitoring to detect inadvertent extreme temperatures (values in the range of 5 to 50 °C), and finally stored at 5 °C.

### Surface microbial testing

Sampling the outer surfaces of holders was done under sterile conditions in a laminar flow biological safety cabinet, where each holder was aseptically removed from the zip lock. The whole outer surface of the holders, including the aluminum and polycarbonate window, was thoroughly sampled with pre-moistened swabs (Swab Rinse Kit SRK, with 10 mL of Letheen Broth, Copan) to improve the uptake of any bacteria present on the dry surfaces. Letheen Broth was incubated with a swab at 30 °C, 80 rpm (Ecotron, Infors HT) for 24 h. Then incubated Letheen Broth was streaked on Columbia (VWR) 5% Sheep Blood Petri 90 mm (0.1 mL per plate). Plates were incubated at 30 °C for at least one week. One to two colonies for each morphotype were taken for genotypic identification through ribosomal RNA (rRNA) sequencing by MicroSEQ (Applied Biosystems) and, for the isolates difficult to identify through 16 S rRNA gene sequencing, additional phenotypic identification by Maldi Biotyper (Bruker) MALDI-TOF mass spectrometry was performed. Both techniques were performed by Confarma France SAS. Clones to be identified through MicroSEQ were sent to Confarma on FTA Cards (Whatman). Basic Local Alignment Tool was processed on MicroSEQ™ ID 16 S rDNA 500 Microbial Library, v2019. Clones to be identified through MALDI-TOF spectrometry were sent on Tryptic Soy Agar (VWR). Mass spectra were assigned using the MALDI Biotyper^®^ 8468 MSP Library.

### Optical microscopy and image analyses

The MATISS sample holders were mounted on a raster X-Y table. A tile scanning mode was applied to image a full glass surface, seen through four windows that are visible across the polycarbonate cover (Supplementary Fig. 1). An optical macroscope MacroFluo Leica Z16 ApoA and a PlanApo 5×/0.5 coupled to a QImaging QICAM fast 1394 camera (12 bits, 1392 × 1040) controlled by a MetaMorph interface was used. A stack of 30 RGB images per window (75 ms exposition time) was produced at low zoom (×3), and of 1452 images (100 ms exposition time) at high zoom (×30). The RGB gain was fixed to 1.19/1/2.31 for the MATISS-1 (LZ) holders and to 1.71/1/2.16 for MATISS-1 (HZ), MATISS-2 et MATISS-2.5 holders. Bayer Method = Average Four.

Processing of the stack of images was applied to produce an output listing for the position, area, and elongation ratio of each particle (Lemelle et al. 2020^45^). For the stack recorded at low zoom, the segmentation of the image was performed on the blue component using a constant threshold value of grey level of about 75 that was empirically determined (Supplementary Fig. 6a). For the stack recorded at high zoom, the images containing macroscopic objects with shadows masking the small particles were removed from the stack. The segmentation was also performed on the blue component using a threshold value equal to the median value (background) incremented by two times the average value of the standard deviation of the intensity of the stack (Supplementary Fig. 6b). All the particle positions were then compiled using the Analyze Particle module of FIJI and crops centered on these positions were sampled in the blue component of the low zoom stack, and in an 8-bit gray scale average of the RGB images of the high zoom stack. The segmentation of every particle in the crops was further refined using a local threshold value that was a function of the mean value of the crops and the area and the elongation of the central particle compiled using the Analyze Particle module of FIJI.

### Reporting summary

Further information on research design is available in the Nature Research Reporting Summary linked to this article.

## Supplementary information

NPJMGRAV_00758_SuppMat_

Reporting Summary Checklist

## Data Availability

The dataset of particle areas analyzed during the current study are available from the corresponding authors on reasonable request.

## Code availability

The 2 codes applied during the current study, that are described in the Supplementary Fig. 6 are available from the corresponding authors on reasonable request.

## References

[1] Ott M, Pierson D (2014). Space habitation and microbiology: status and roadmap of space agencies. Microbes Environ..

[2] International Space Exploration Coordinating Group, The Global Exploration Roadmap, ISECG Technical Report, Jan. https://www.globalspaceexploration.org/wordpress/wp-content/isecg/GER_2018_small_mobile.pdf (2018).

[3] Yamaguchi N (2014). Microbial monitoring of crewed habitats in space—current status and future perspectives. Microbes Environ..

[4] Baranov VM (2006). Main results of the Biorisk experiment on the International Space Station. Aviakosm. Ekol. Med..

[5] Jorgensen JH (1997). Development of an antimicrobial susceptibility testing method suitable for performance during space flight. J. Clin. Microbiol..

[6] Farkas Á, Farkas G (2021). Effects of Spaceflight on Human Skin. Ski. Pharmacol. Physiol..

[7] LaPelusa M (2021). Microbiome for Mars: surveying microbiome connections to healthcare with implications for long-duration human spaceflight, virtual workshop, July 13, 2020. Microbiome.

[8] Santomartino R (2020). No effect of microgravity and simulated Mars gravity on final bacterial cell concentrations on the International Space Station: applications to space bioproduction. Front. Microbiol..

[9] Zea L (2020). Potential biofilm control strategies for extended spaceflight missions. Biofilm.

[10] Wilson JW (2007). Space flight alters bacterial gene expression and virulence and reveals a role for global regulator Hfq. Proc. Natl Acad. Sci..

[11] Wilson JW (2008). Media ion composition controls regulatory and virulence response of Salmonella in spaceflight. PloS One.

[12] Zea L (2017). Phenotypic changes exhibited by *E. coli* cultured in space. Front. Microbiol..

[13] Mukhopadhyay S, Bagh S (2020). A microgravity responsive synthetic genetic device in *Escherichia coli*. Biosens. Bioelectron..

[14] Bijlani S, Stephens E, Singh NK, Venkateswaran K, Wang CCC (2021). Advances in space microbiology. iScience.

[15] Green MJ, Aylott JW, Williams P, Ghaemmaghami AM, Williams PM (2021). Immunity in space: prokaryote adaptations and immune response in microgravity. Life.

[16] Fajardo-Cavazos P, Nicholson WL (2021). Mechanotransduction in prokaryotes: a possible mechanism of spaceflight adaptation. Life.

[17] Huang B, Li D-G, Huang Y, Liu C-T (2018). Effects of spaceflight and simulated microgravity on microbial growth and secondary metabolism. Mil. Med. Res..

[18] Bauer J (2020). Microgravity and Cell Adherence. Int. J. Mol. Sci..

[19] Vaishampayan A, Grohmann E (2019). Multi-resistant biofilm-forming pathogens on the International Space Station. J. Biosci..

[20] Lin X (2020). The impact of spaceflight and simulated microgravity on cell adhesion. Int. J. Mol. Sci..

[21] Acres JM, Youngapelian MJ, Nadeau J (2021). The influence of spaceflight and simulated microgravity on bacterial motility and chemotaxis. npj Microgravity.

[22] Novikova N (2006). Survey of environmental biocontamination on board the International Space Station. Res. Microbiol..

[23] Lang JM (2017). A microbial survey of the International Space Station (ISS). PeerJ.

[24] Ott CM, Bruce RJ, Pierson DL (2004). Microbial characterization of free floating condensate aboard the Mir Space Station. Microb. Ecol..

[25] Ichijo T, Hieda H, Ishihara R, Yamaguchi N, Nasu M (2013). Bacterial monitoring with adhesive sheet in the international space station-“Kibo”, the Japanese experiment module. Microbes Environ..

[26] Ichijo T, Shimazu T, Nasu M (2020). Microbial monitoring in the International Space Station and its application on Earth. Biol. Pharm. Bull..

[27] Mahnert A (2021). Microbiome dynamics during the HI-SEAS IV mission, and implications for future crewed missions beyond Earth. Microbiome.

[28] Ichijo T, Yamaguchi N, Tanigaki F, Shirakawa M, Nasu M (2016). Four-year bacterial monitoring in the International Space Station-Japanese experiment module ‘Kibo’ with culture-independent approach. NPJ Microgravity.

[29] Pierson DL (2001). Microbial contamination of spacecraft. Gravit. Space Biol. Bull..

[30] Buchovec I, Gricajeva A, Kalėdienė L, Vitta P (2020). Antimicrobial photoinactivation approach based on natural agents for control of bacteria biofilms in spacecraft. Int. J. Mol. Sci..

[31] Zea L (2018). Design of a spaceflight biofilm experiment. Acta Astronaut..

[32] Morrison MD (2021). Investigation of spaceflight induced changes to astronaut microbiomes. Front. Microbiol..

[33] Tirumalai MR (2017). The adaptation of *Escherichia coli* cells grown in simulated microgravity for an extended period is both phenotypic and genomic. npj Microgravity.

[34] Balistreri, S. F. S., Steele, J. W., Caron, M. E. & Laliberte, Y. J., International space station common cabin air assembly condensing heat exchanger hydrophilic coating operation, recovery, and lessons learned. NASA Technical Report JSC-CN-27469. https://ntrs.nasa.gov/archive/nasa/casi.ntrs.nasa.gov/20130000766.pdf (2013).

[35] James, J. T., Parmet, A. J. & Pierson, D. L. Aerospace toxicology and microbiology. https://ntrs.nasa.gov/citations/20070032022 (2007).

[36] Novikova ND (2004). Review of the knowledge of microbial contamination of the Russian manned spacecraft. Microb. Ecol..

[37] Otter JA, Yezli S, French GL (2011). The role played by contaminated surfaces in the transmission of nosocomial pathogens. Infect. Control. Hosp. Epidemiol..

[38] Otter JA (2016). Transmission of SARS and MERS coronaviruses and influenza virus in healthcare settings: the possible role of dry surface contamination. J. Hosp. Infect..

[39] Weber D, Anderson D, Rutala W (2013). The role of the surface environment in healthcare-associated infections. Curr. Opin. Infect. Dis..

[40] Siegel JD, Rhinehart E, Jackson M, Chiarello L, Health care infection control practices advisory committee. (2007). Guideline for isolation precautions: preventing transmission of infectious agents in health care settings. Am. J. Infect. Control.

[41] Ott, C. M. *Risk of adverse health effects due to host-microorganism interactions*. https://ntrs.nasa.gov/search.jsp?R=20170001973 (2016).

[42] Smirnov, E. M., Ivanov, N. G., Telnov, D. S., Son, C. H. & Aksamentov, V. K. *Computational Fluid Dynamics study of air flow characteristics in the Columbus Module*, SAE Technical Paper 2004-01-2500. 10.4271/2004-01-2500 (2004).

[43] Sethi SK, Manik G (2018). Recent progress in super hydrophobic/hydrophilic self-cleaning surfaces for various industrial applications: a review. Polym. Plast. Technol. Eng..

[44] Salmela A (2020). Measurement and simulation of biocontamination in an enclosed habitat. Aerosol Sci. Eng..

[45] Lemelle L (2020). Towards a passive limitation of particle surface contamination in the Columbus module (ISS) during the MATISS experiment of the Proxima Mission. npj Microgravity.

[46] Moazzam P, Razmjou A, Golabi M, Shokri D, Landarani-Isfahani A (2016). Investigating the BSA protein adsorption and bacterial adhesion of Al-alloy surfaces after creating a hierarchical (micro/nano) superhydrophobic structure. J. Biomed. Mater. Res..

[47] Mandal P, Ivvala J, Arora HS, Ghosh SK, Grewal HS (2022). Bioinspired micro/nano structured aluminum with multifaceted applications. Colloids Surf. B: Biointerfaces.

[48] Li J (2021). Facile Li-Al layered double hydroxide films on Al alloy for enhanced hydrophobicity, anti-biofouling and anti-corrosion performance. J. Mater. Sci. Technol..

[49] Lee Y (2020). Lubricant-infused directly engraved nano-microstructures for mechanically durable endoscope lens with anti-biofouling and anti-fogging properties. Sci. Rep..

[50] Jiang R (2020). Lotus-leaf-inspired hierarchical structured surface with non-fouling and mechanical bactericidal performances. Chem. Eng. J..

[51] Kefallinou D (2020). Optimization of Antibacterial Properties of “Hybrid” Metal-Sputtered Superhydrophobic Surfaces. Coatings.

[52] Wang T (2020). Robust Biomimetic Hierarchical Diamond Architecture with a Self-Cleaning, Antibacterial, and Antibiofouling Surface. ACS Appl. Mater. Interfaces.

[53] Grinenval E, Nonglaton G, Vinet F (2014). Spatially controlled immobilisation of biomolecules: a complete approach in green chemistry. Appl. Surf. Sci..

[54] Sadri B (2018). Wearable and implantable epidermal paper-based electronics. ACS Appl. Mater. Interfaces.

[55] Glavan AC (2013). Rapid fabrication of pressure-driven open-channel microfluidic devices in omniphobic RF paper. Lab Chip.

[56] Sala de Medeiros M, Chanci D, Moreno C, Goswami D, Martinez RV (2019). Waterproof, Breathable, and Antibacterial Self-Powered e-Textiles Based on Omniphobic Triboelectric Nanogenerators. Adv. Funct. Mater..

[57] Chen Y (2018). Superhydrophobic coatings on gelatin-based films: fabrication, characterization and cytotoxicity studies. RSC Adv..

[58] Sabino RM, Kauk K, Movafaghi S, Kota A, Popat KC (2019). Interaction of blood plasma proteins with superhemophobic titania nanotube surfaces. Nanomed.: Nanotechnol., Biol. Med..

[59] Silverio V, Canane PAG, Cardoso S (2019). Surface wettability and stability of chemically modified silicon, glass and polymeric surfaces via room temperature chemical vapor deposition. Colloids Surf. A: Physicochem. Eng..

[60] Yang Y, Bittner AM, Baldelli S, Kern K (2008). Study of self-assembled triethoxysilane thin films made by casting neat reagents in ambient atmosphere. Thin Solid Films.

[61] Pellerite MJ, Wood EJ, Jones VW (2002). Dynamic contact angle studies of self-assembled thin films from fluorinated alkyltrichlorosilanes. J. Phys. Chem. B.

[62] Madigan, M. & Martinko, J. *Microorganisms and microbiology*. Brock Biology of Microorganisms, 11th ed. Upper Saddle River, New Jersey (NJ): Pearson Prentice Hall, 1–20 (2006).

[63] Willey, J. M. Sherwood, L. M. & Woolverton C. J. *Prescott’s Microbiology*, 7th Edition, Mc Graw-Hill Companies Inc. (2008).

[64] Busse HJ (2016). Review of the taxonomy of the genus *Arthrobacter*, emendation of the genus *Arthrobacter sensu lato*, proposal to reclassify selected species of the genus *Arthrobacter* in the novel genera *Glutamicibacter gen. nov., Paeniglutamicibacter gen. nov., Pseudoglutamicibacter gen. nov., Paenarthrobacter gen. nov. and Pseudarthrobacter gen. nov*., and emended description of *Arthrobacter roseus*. Int. J. Syst. Evol. Microbiol..

[65] Patel S, Gupta RS (2020). A phylogenomic and comparative genomic framework for resolving the polyphyly of the genus Bacillus: Proposal for six new genera of Bacillus species, *Peribacillus gen. nov., Cytobacillus gen. nov*., *Mesobacillus gen. nov*., *Neobacillus gen. nov., Metabacillus gen. nov. and Alkalihalobacillus gen. nov*. Int. J. Syst. Evol. Microbiol..

[66] Chu, Z. et al. Smart Superhydrophobic Films with Self-Sensing and Anti-Icing Properties Based on Silica Nanoparticles and Graphene. *Adv. Mater. Interfaces*. **7** (2020).

[67] Kim M-K (2015). Enhanced Jumping-Droplet Departure. Langmuir.

[68] Boreyko JB, Chen C-H (2009). Self-propelled dropwise condensate on superhydrophobic surfaces. Phys. Rev. Lett..

[69] Watson GS (2015). Removal mechanisms of dew via self-propulsion off the gecko skin. J. R. Soc. Interface.

[70] Watson GS, Gellender M, Watson JA (2014). Self-propulsion of few drops on lotus leaves: a potential mechanism for self cleaning. Biofouling.

