# Supplementary material for: Passive limitation of surface contamination by perFluoroDecylTrichloroSilane coatings in the ISS during the MATISS experiments
Source: NPJ Microgravity. 2022 Aug 4;8:31. doi: 10.1038/s41526-022-00218-3 (PMC9352769; doi:10.1038/s41526-022-00218-3)
Supplement: Supplementary file 1 — NPJMGRAV_00758_SuppMat_ [file 41526_2022_218_MOESM1_ESM.pdf]

Supplementary Materials

Passive limitation of surface contamination by perFluoroDecylTrichloroSilane coatings in the ISS during the MATISS experiments

Laurence Lemelle, Sébastien Rouquette, Eléonore Mottin, Denis Le Tourneau, Pierre R. Marcoux, Cécile Thévenot, Alain Maillet, Guillaume Nonglaton, Christophe Place

Supplementary Figure 1 – Photograph of two sample holders of MATISS-2 (p. 2)
Supplementary Figure 2 – Surface contamination on FDTS coating (p.3)
Supplementary Figure 3 – Surface contamination by coarse particles (p. 4-5)
Supplementary Figure 4 – Surface contamination by fine particles (p. 6-8)
Supplementary Figure 5 – Schematic representation kinetics of the location of particles on the control and FDTS coating (p. 9)
Supplementary Figure 6 – Flowcharts of the processing of the stacks of images (p. 10-11)

Supplementary Table 1 – Taxonomy of isolates based on the identification by rRNA sequencing analysis, completed by MALDI-TOF MS (p. 12)
Supplementary Table 2 – Occupancy rates (p. 13)

Supplementary Figure 1: Photograph of two sample holders of the MATISS-2 campaign installed next to the Return Grid Sensor Housing (RGSH) in the port-side cone of the Columbus module of the ISS. The FDTS lamellae (red frame) were mounted in the holders to have one side closest to the air entrance. Scale bar is 10cm. Photograph courtesy of NASA/ESA permissible to use within the public domain.

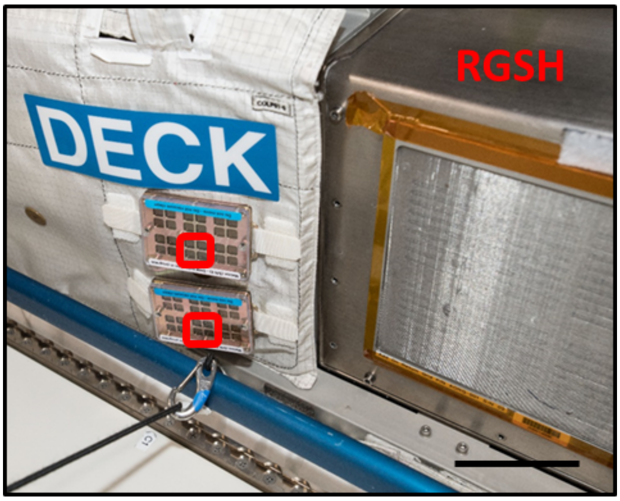

Supplementary Figure 2: Surface contamination on FDTS coating. (a) Cumulative particle size function of coarse particles (50µm² < Area < 1500µm²) in percentage per Area unit for 193 days of exposure in MATISS-1 (red), 354 days in MATISS-2 (blue) and 365 days MATISS-2.5 (green). Error bars areevaluated here as the standard deviation for each area fractions measured from at least four different windows for each campaign. Statistical differences between distributions of the different campaigns were examined by unpaired Student's *t*-test and not found to be significantly different (*p*-values > 0.3). **(b)** Cumulative particle size function of fine particles (0.5µm² < Area < 50µm²) in percentage per Area unit for 193 days (red, MATISS-1), 354 days (blue, MATISS-2) and 365 days (green, MATISS-2.5) of exposure. Error bars are evaluated here as the standard deviation for each area fractions measured from at least four different windows for each campaign. The statistical differences between fine particle distributions were found to be significantly different between the MATISS-1 distribution and MATISS-2 or MATISS-2.5 (****p*-values < 0.001).

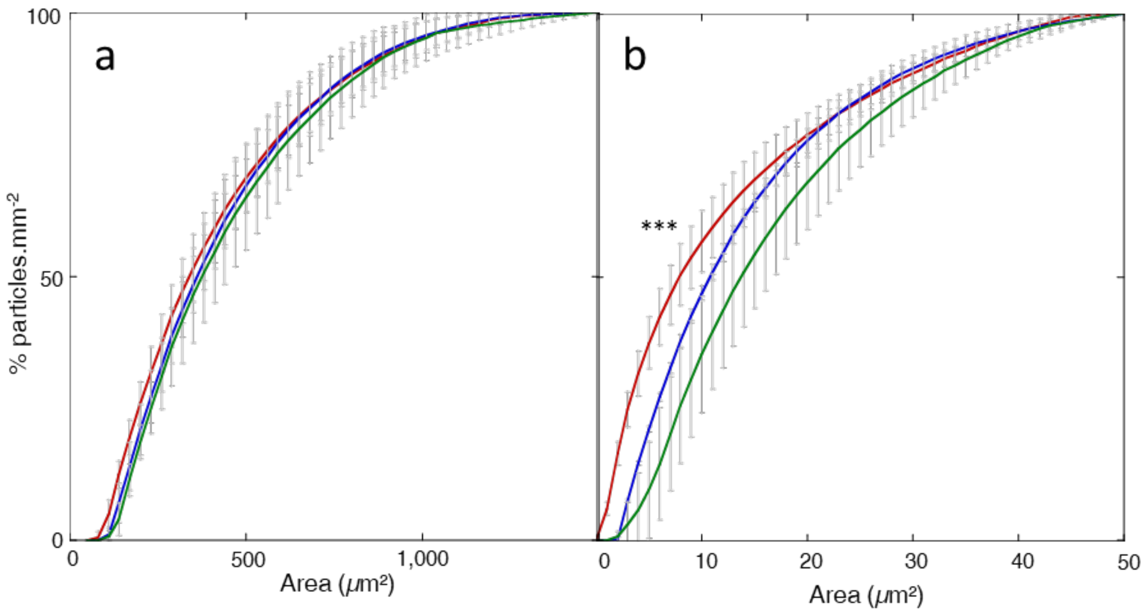

Supplementary Figure 3: Surface contamination by coarse particles ($50\mu m^2 < Area < 1500\mu m^2$) **(a)** Mosaic of optical images displaying one single particle from the MATISS-2, with circularity (4.Π.Area / Perimeter^2) values in the range of 0.8 to 1. **(b)** Mosaic of optical images displaying one single particle from the MATISS-2, with circularity values lower than 0.8 **(c)** Row of optical images displaying one single particle from the MATISS-2.5. Scale bar is 10µm.

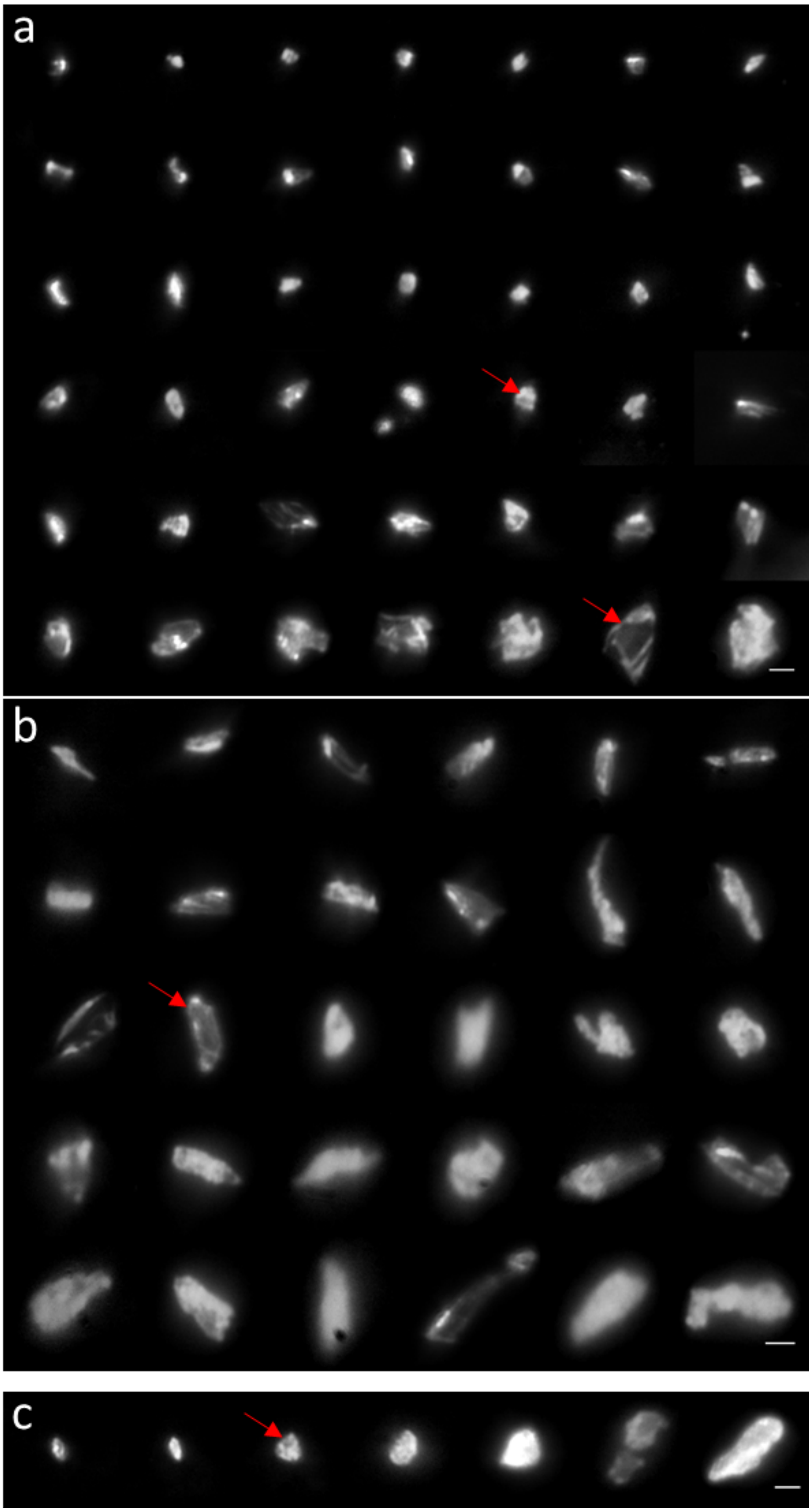
a
b
c

Supplementary Figure 4: Surface contamination by fine particles. **(a)** Mosaic of optical images displaying one single particle with an area higher than 0.5µm² and lower than 5µm². **(b)** Mosaic of optical images displaying one single particle with an area higher than 5µm² and lower than 20µm². **(c)** Mosaic of optical images displaying one single particle with an area higher than 20µm² and lower than 50µm². Scale bar is 2µm. Rows 1-6 show particles from the MATISS-2 campaign, the row 7 shows particles from the MATISS-2.5 campaign.

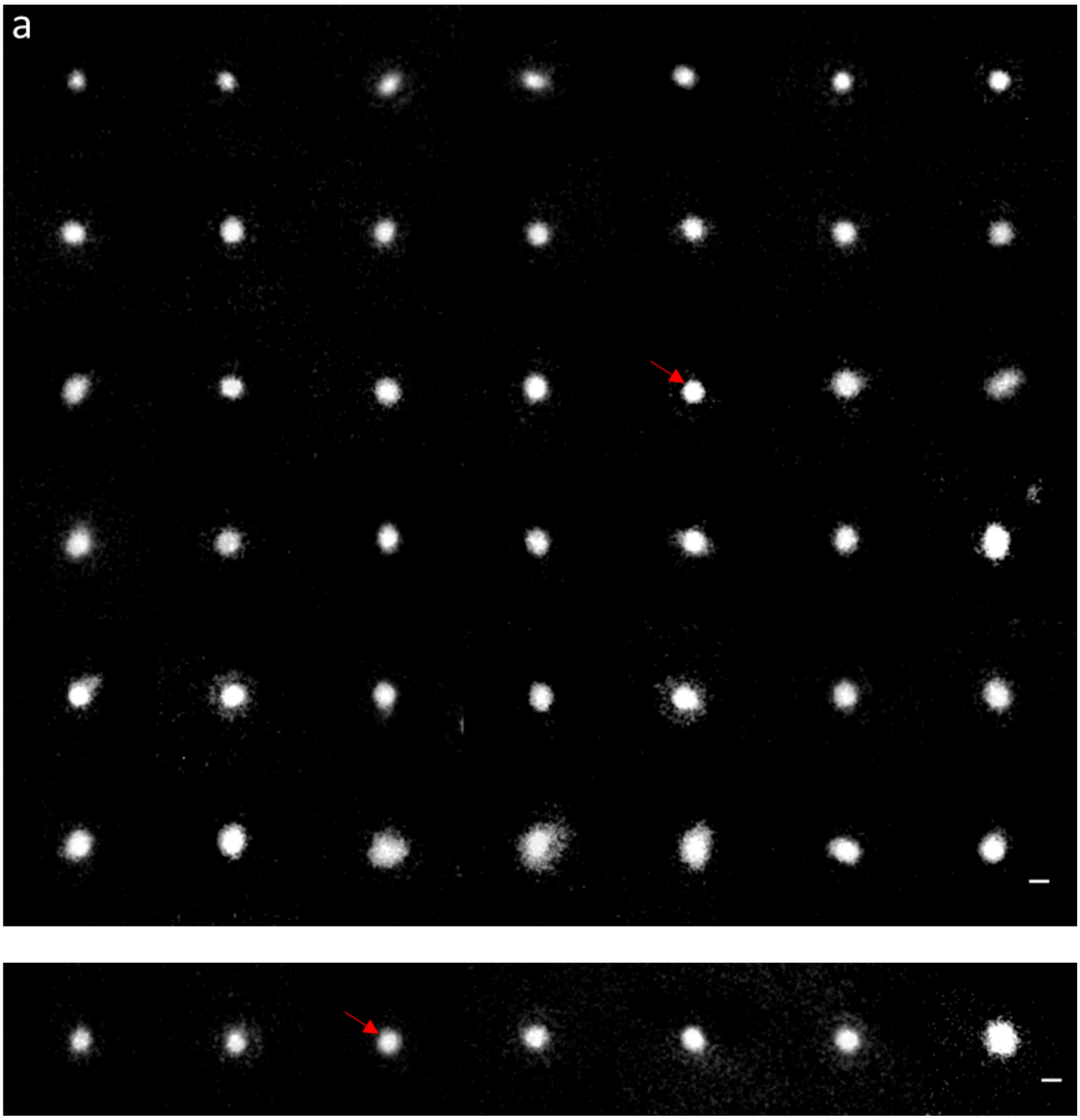

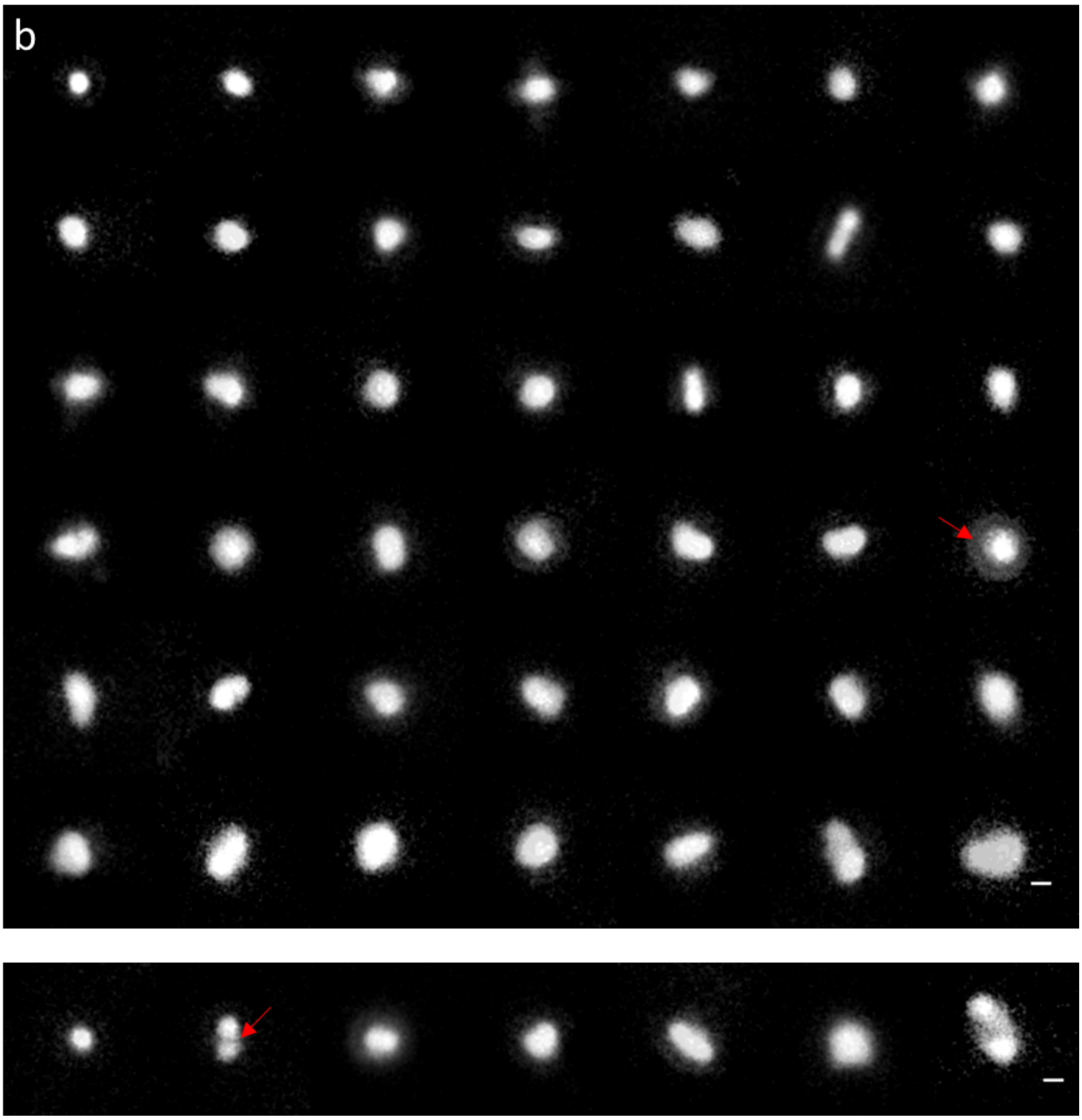
b

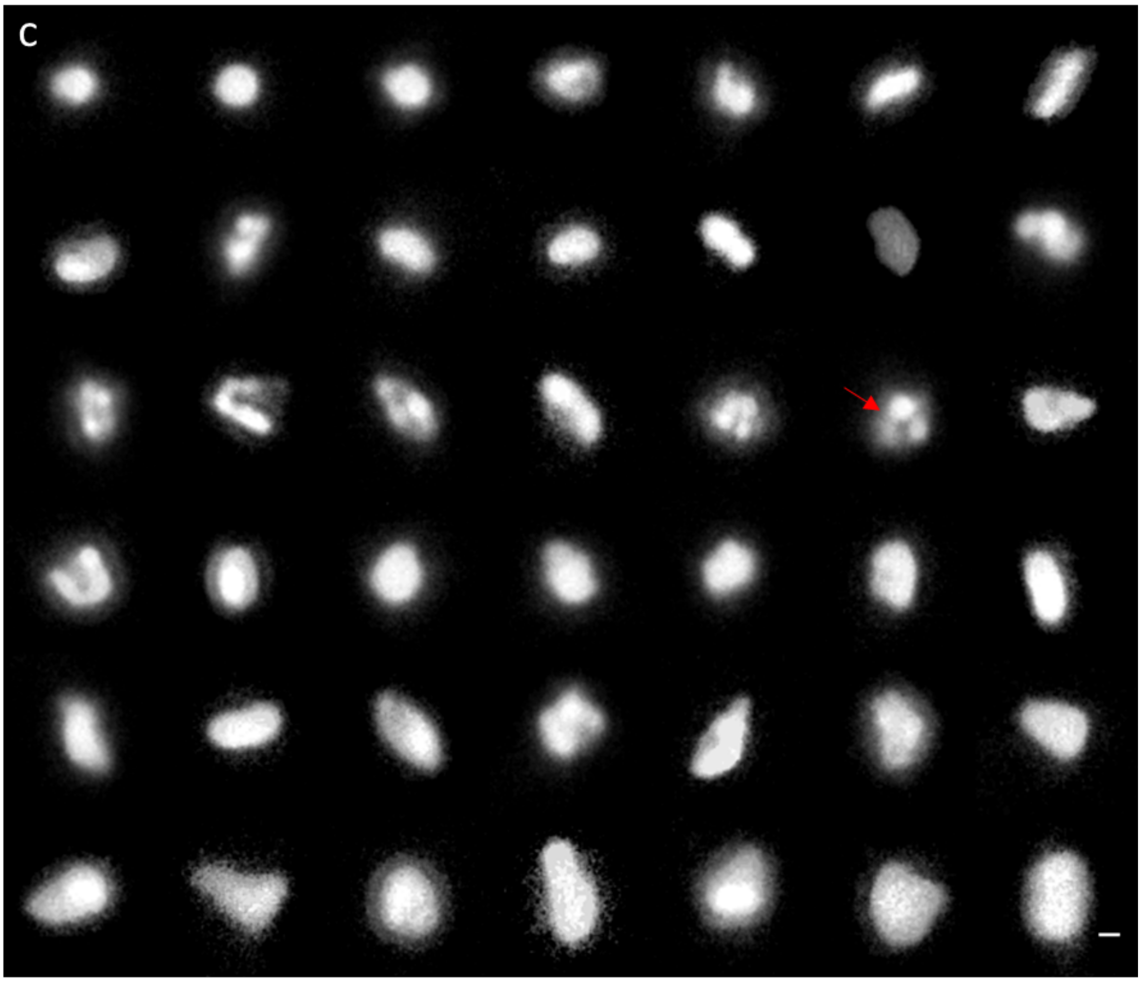
C

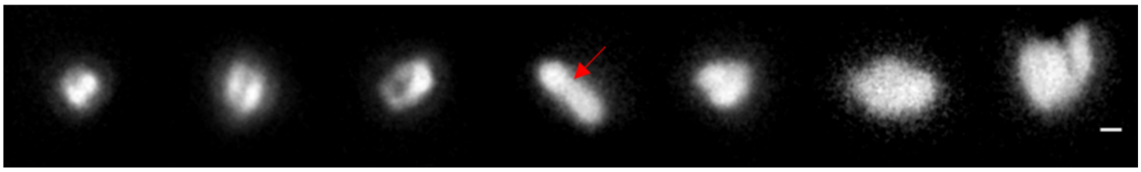

Supplementary Figure 5: Schematic representation kinetics of the location of particles on the control and FDTS coating. (a) Location of the coarse particles with different windows exposed at 41 days (left), 95 days (middle), and 354 days (right). The side near the closest aperture is in y position =0. Each particle is represented by a circle whose color (yellow/orange/violet scale from 50 to 500µm²) and size (ratio 1 to 1.5 from 50 to 1500µm²) are proportional to the particle surface. The particles number histogram versus the y position (vertical axis) of particles on surfaces (right). **(b)** Same representation for fine particles with a yellow/orange/violet scale from 0 to 50µm² and size ratio of 1 to 1.5 for 0 to 50µm² particle surfaces.

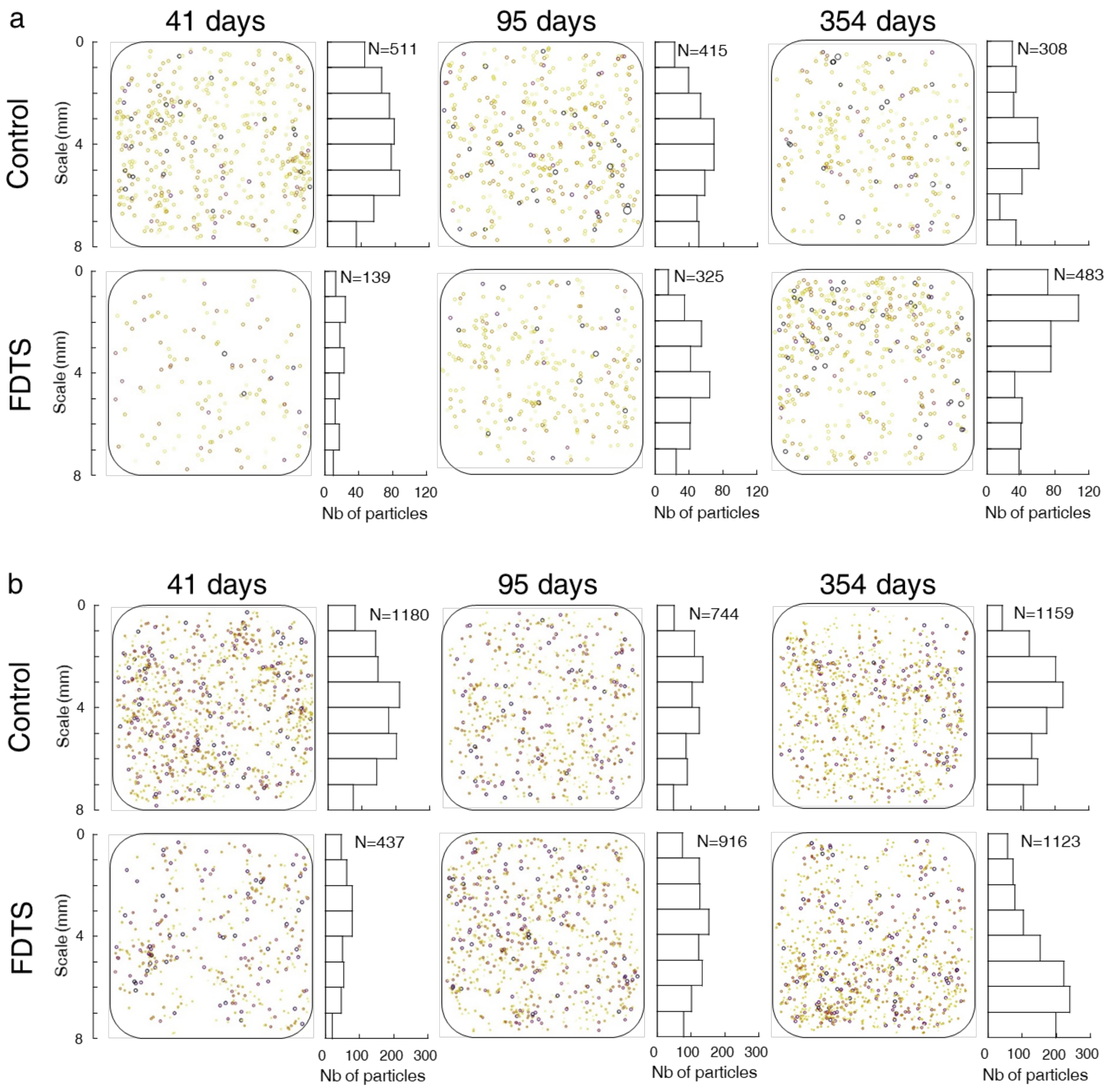

Supplementary Figure 6: Flowcharts of the processing of the stacks of images. (a) Flowchart for the images acquired at low zoom. (b) Flowchart for the images acquired at high zoom.

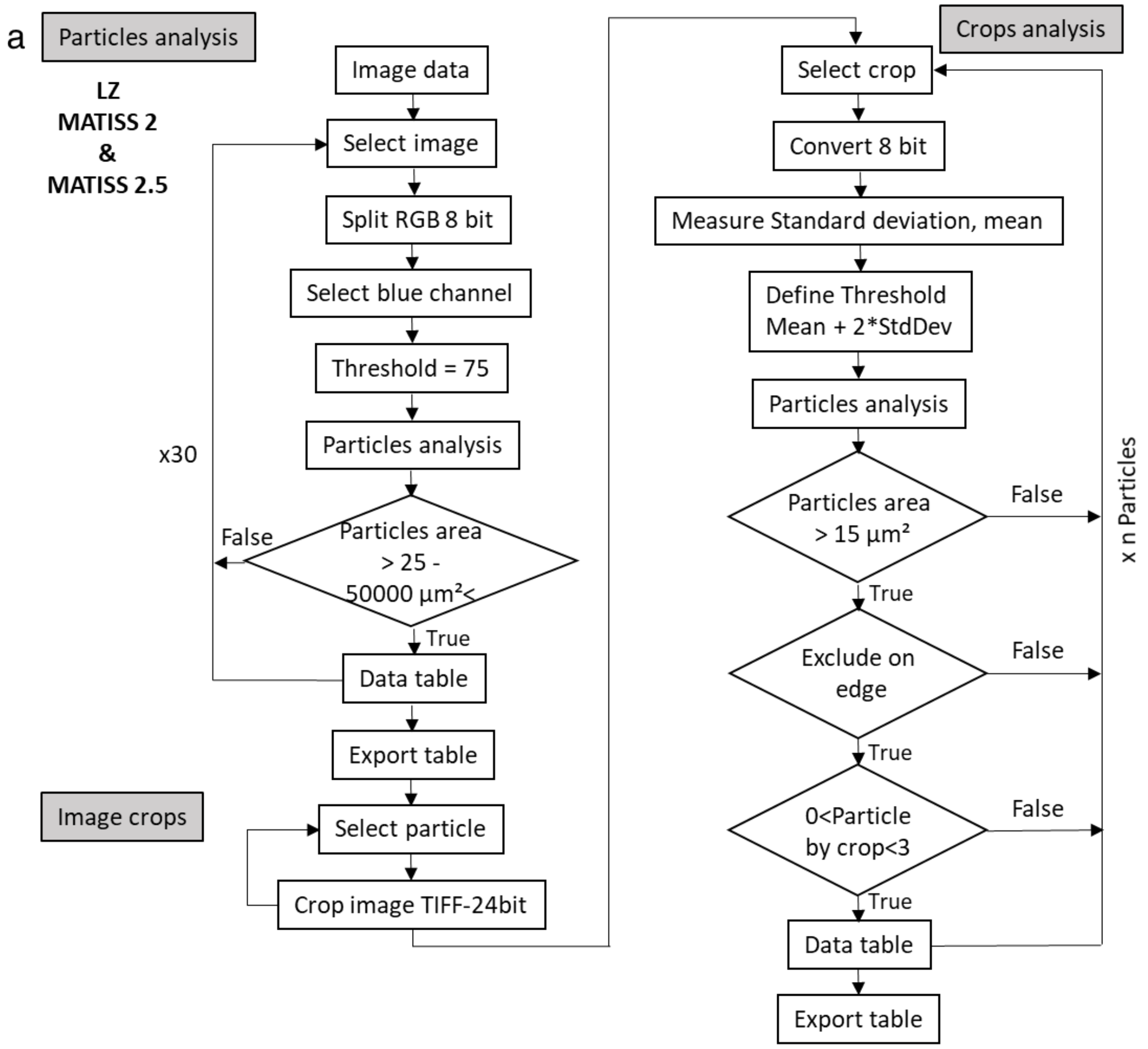

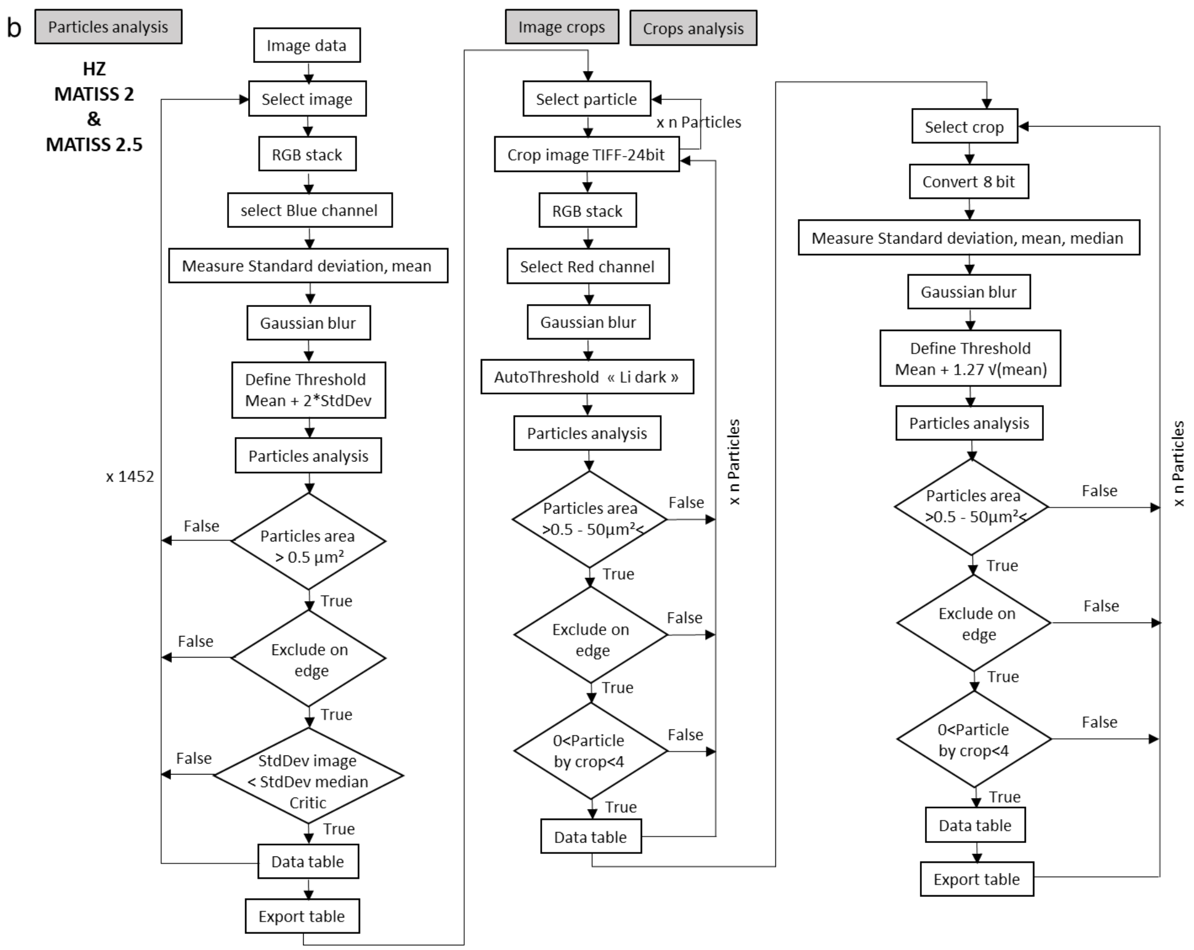
b
Particles analysis
HZ
MATISS 2
&
MATISS 2.5
Image data
Select image
RGB stack
select Blue channel
Measure Standard deviation, mean
Gaussian blur
Define Threshold
Mean + 2*StdDev
Particles analysis
x 1452
Particles area
> 0.5 µm²
False
True
Exclude on edge
False
True
StdDev image
< StdDev median
Critic
False
True
Data table
Export table
Image crops
Crops analysis
Select particle
x n Particles
Crop image TIFF-24bit
RGB stack
Select Red channel
Gaussian blur
AutoThreshold « Li dark »
Particles analysis
Particles area
>0.5 - 50µm²<
False
True
Exclude on edge
False
True
0<Particle
by crop<4
False
True
Data table
x n Particles
Select crop
Convert 8 bit
Measure Standard deviation, mean, median
Gaussian blur
Define Threshold
Mean + 1.27 √(mean)
Particles analysis
Particles area
>0.5 - 50µm²<
False
True
Exclude on edge
False
True
0<Particle
by crop<4
False
True
Data table
Export table
x n Particles

Supplementary Table 1: Taxonomy of isolates based on the identification by rRNA sequencing analysis, completed by MALDI-TOF MS. Confidence level is abbreviated here as CL. Because of the culture in Letheen Broth prior to streaking with Columbia agar, the isolated genera and species are provided without giving some occurrence.

Phylum/order/class	Family	Genus	Species	CL*	MALDI-TOF
**16S rRNA gene analysis**					
Firmicutes/ Bacillales/ Bacilli	*Staphylococcaceae*	*Staphylococcus*	*epidermidis*	Species	
	Staphylococcaceae	*Staphylococcus*	*hominis*	Species	
	Staphylococcaceae	*Staphylococcus*	*epidermidis*	Genus. Multiple species, possibly *S. caprae*	*S. epidermidis* CL: species
	Bacillaceae	*Bacillus*	*horneckiae*	Most closely related to *Bacillus sp.*	*B. horneckiae* CL: species
	Bacillaceae	*Bacillus*	Possibly *subtilis*	Genus. Multiple species, possibly *B. mojavensis* or *B. vallismortis*	
	Bacillaceae	*Bacillus*	Possibly *simplex*	Genus. Multiple species, possibly *B. butanolivorans* or *B. muralis*	*B. simplex* CL: genus
	Paenibacillaceae	Possibly *Paenibacillus sp.*		Most closely related to *Paenibacillus sp.*	
Actinobacteria/ Micrococcales/ Actinomycetia	*Micrococcaceae*	*Micrococcus*	*luteus*	Species	
	Micrococcaceae	Most closely related to *Pseudarthrobacter sp.*		Multiple genera, possibly *Arthrobacter pascens*	*Pseudarthrobacter sp.*, possibly *polychromogenes*, *scleromae* or *oxydans* CL: genus
Proteobacteria Pseudomonadales/ Gammaproteobacteria	*Pseudomonadaceae*	*Pseudomonas*	*fulva*	Species	

Supplementary Table 2: Occupancy rates of the Columbus Module by the European astronauts, η (%), evaluated as the period the EU astronauts stayed in the ISS during the exposure period of a MATISS holder (one line per exposure period), the periods being defined here from the dates of the beginning and the end of the exposure and the astronaut's stay.

	Begin	End	N (days)	Astronauts	Begin	End	N (days)	η (%)	θ LZ (particles.mm^{-2})	θ LZ (particles.mm^{-2} by month)	θ HZ (particles.mm^{-2})	θ HZ (particles.mm^{-2} by month)
**MATISS-1**	20/11/16	01/06/17	193	T. Pesquet	20/11/16	01/06/17	193	100	2.45	0.38	4.45	0.69
**MATISS-2**	23/08/18	03/10/18	41	A. Gerst	23/08/18	03/10/18	41	100	0.58	0.42	1.82	1.33
	23/08/18	26/11/18	95	A. Gerst	23/08/18	26/11/18	95	100	1.35	0.43	3.82	1.21
	23/08/18	20/12/18	119	A. Gerst	23/08/18	12/08/19	354	34	2.01	0.17	4.68	0.40
**MATISS-2.5**												
**Borosilicate**	25/09/19	06/02/20	134	L. Parmitano	25/09/19	24/09/20	365	37	0.31	0.03	0.50	0.04
**Suprasil**	25/09/19	06/02/20	134	L. Parmitano	25/09/19	24/09/20	365	37	0.40	0.03	1.53	0.13
